# Epithelial-Mesenchymal Transition (EMT): The Type-2 EMT in Wound Healing, Tissue Regeneration and Organ Fibrosis

**DOI:** 10.3390/cells10071587

**Published:** 2021-06-23

**Authors:** Guya D. Marconi, Luigia Fonticoli, Thangavelu Soundara Rajan, Sante D. Pierdomenico, Oriana Trubiani, Jacopo Pizzicannella, Francesca Diomede

**Affiliations:** 1 Department of Medical, Oral and Biotechnological Sciences, University “G. d’Annunzio” Chieti-Pescara, 66100 Chieti, Italy; guya.marconi@unich.it; 2 Department of Innovative Technologies in Medicine & Dentistry, University “G. d’Annunzio” Chieti-Pescara, 66100 Chieti, Italy; luigia.fonticoli@unich.it (L.F.); sante.pierdomenico@unich.it (S.D.P.); 3 Department of Biotechnology, School of Life Sciences, Karpagam Academy of Higher Education, Coimbatore 641021, India; tsrajanpillai@gmail.com; 4 ASL02 Lanciano-Vasto-Chieti, “Ss. Annunziata” Hospital, 66100 Chieti, Italy; jacopo.pizzicannella@unich.it

**Keywords:** wound healing, fibroblasts, epithelial mesenchymal transition, tissue regeneration, fibrosis, inflammation

## Abstract

The epithelial–mesenchymal transition (EMT) is an essential event during cell development, in which epithelial cells acquire mesenchymal fibroblast-like features including reduced intercellular adhesion and increased motility. EMT also plays a key role in wound healing processes, which are mediated by inflammatory cells and fibroblasts. These cells secrete specific factors that interact with molecules of the extracellular matrix (ECM) such as collagens, laminins, elastin and tenascins. Wound healing follows four distinct and successive phases characterized by haemostasis, inflammation, cell proliferation and finally tissue remodeling. EMT is classified into three diverse subtypes: type-1 EMT, type-2 EMT and type-3 EMT. Type-1 EMT is involved in embryogenesis and organ development. Type-2 EMT is associated with wound healing, tissue regeneration and organ fibrosis. During organ fibrosis, type-2 EMT occurs as a reparative-associated process in response to ongoing inflammation and eventually leads to organ destruction. Type-3 EMT is implicated in cancer progression, which is linked to the occurrence of genetic and epigenetic alterations, in detail the ones promoting clonal outgrowth and the formation of localized tumors. The current review aimed at exploring the role of EMT process with particular focus on type-2 EMT in wound healing, fibrosis and tissue regeneration, as well as some recent progresses in the EMT and tissue regeneration field, including the modulation of EMT by biomaterials.

## 1. Introduction

The epithelial–mesenchymal transition (EMT) is a pivotal process that plays a key role in physiological and pathological events, such as embryogenesis, wound healing and cancer development [1]. EMT allows the cells to switch from an epithelial state to a mesenchymal one [2]. The opposite process, mesenchymal–epithelial transition (MET), can analogously produce epithelial from mesenchymal cells.

During EMT, a polarized epithelial cell, which normally interacts with the basement membrane via its basal surface, undergoes multiple biochemical changes resulting in the acquisition of a mesenchymal phenotype. The mesenchymal phenotype includes enhanced migratory capacity, invasiveness, and elevated resistance to apoptosis and a greatly increased production of extracellular matrix (ECM) components [3].

The literature describes three different types of EMTs, each occurring in a different biological scenario and produce diverse functional results [4].

Type-1 EMT is associated with embryo implantation and development, as well as multiple organ formation, neither provokes fibrosis nor induces an invasive phenotype. This EMT is required for the production of mesenchymal cells (primary mesenchyme) that are capable of successively undergoing a MET to create secondary epithelia. Embryonic development, also known as embryogenesis, is a complex event where both EMT and MET are needed for the final differentiation of specialized cell types and for the formation of the three-dimensional organization of the organs. Cells of primary mesenchyme evidence increased migratory characteristics. At the biochemical level, the EMT correlated with gastrulation is coordinated by canonical Wnt signaling. EMT associated with gastrulation is regulated by Snail, Eomesodermin (Eomes), and Mesoderm posterior protein (Mesps) transcription factors and also by Wnts, which collaborates with Fibroblast Growth Factor (FGF) receptors for regulating EMT linked to the gastrulation. Snail suppresses E-cadherin and promotes EMT mediated by cell adhesion molecules as occludins and claudins and by polarity genes, such as Discs large (Dlg) and Crumbs homolog 3 (Crb3) [5]. During embryonic formation, an EMT including the epithelial cells of the neuroectoderm gives origin to migratory neural crest cells [6]. The EMTs associated with wound healing, tissue regeneration and organ fibrosis are classified as type-2 EMTs. Type-2 EMT events occur as part of a repair-associated process where the epithelial cells differentiate into novel fibroblast-like cells in order to rebuild tissues following trauma and inflammatory damage. These cells evidence epithelial-specific morphology and molecular markers, such as cytokeratin and E-cadherin, but at the same time express the ferroptosis suppressor protein 1 (FSP1, an S100 class of cytoskeletal protein) mesenchymal marker and alpha smooth muscle actin (α-SMA). Type-2 EMTs are associated with inflammation and terminated once repair is completed and inflammation is reduced.

Fibrosis is distinguished by an excess deposition of fibrous connective tissue in an organ. Defined by the pathological accumulation of ECM components, that lead with time to scar tissue development and finally organ dysfunction and failure [7]. Type-2 EMT is linked with tissue repair responses such as fibrosis. To heal damaged tissues, type-2 EMT gives origin to myofibroblasts from epithelia; the healing event is considered as reparative fibrosis if the injury is moderate and acute. Instead, in ongoing chronic inflammation, abnormal formation of myofibroblasts provoke a progressive fibrosis that leads to organ parenchymal destruction due to an excessive ECM deposition.

Thus, tissue fibrosis is an unceasing sort of wound healing resulting from aberrant inflammation process [8].

FSP1, α-SMA and collagen I have provided reliable markers to characterize the mesenchymal products generated by the EMTs that occur during the development of fibrosis in various organs. These markers, along with discoidin domain receptor tyrosine kinase 2 (DDR2), vimentin and desmin, have been used to identify epithelial cells of the kidney, liver, lung and intestine that are in the middle of undergoing an EMT associated with chronic inflammation.

Various in vitro and in vivo studies have reported that EMT is involved in the fibrogenesis of critical organs, such as the kidney, liver, lung, and intestine [9]. For example, studies carried out in a transgenic mouse model have confirmed the involvement of EMT in renal fibrosis and demonstrated that >30% of novel fibroblasts originate from local EMT [10].

Type-3 EMT occurs in neoplastic cells that have experienced genetic and epigenetic modifications, particularly in genes that promote the formation of localized tumours.

Several studies have demonstrated that carcinoma cells can acquire a mesenchymal phenotype and express mesenchymal markers such as α-SMA, FSP1, vimentin and desmin [11]. Such cells continue to express epithelial markers, but novel mesenchymal markers have already been obtained [12].

**Figure 1:**
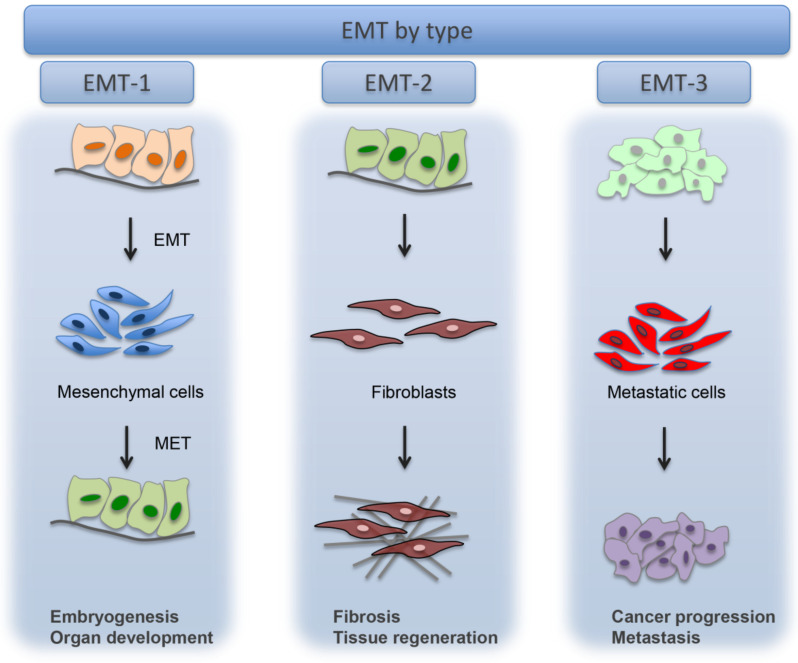
Types of EMT. Type-1 EMT, Type-2 EMT, Type-3 EMT. Reprinted from Zeisberg, M.; Neilson, E.G. Biomarkers for epithelial-mesenchymal transitions. *J. Clin. Invest.* **2009**, *119*, 1429–1437 [4].

These alterations, which modify oncogenes and tumour suppressor genes, cooperate with the EMT regulatory circuitry to induce different effects from those reported in type-1 and type-2 EMTs. For instance, the migratory cancer cells produced by type-3 EMT promote secondary tumours in distant tissues with epithelial phenotypes. This suggests that the reversibility of EMT, which is a crucial factor in embryogenesis, also plays a key role in the development of secondary metastatic nodules [13]. Therefore, type-3-EMT is necessary for enabling metastatic cancer cells to escape apoptosis and induce the expression of oncogenes [14] (Figure 1).

Over the past two decades, emerging studies have reported that damaged epithelial cells may act as crucial sources of fibroblasts and contribute to organ fibrosis through type-2 EMT [2]. Organ fibrosis, which happens in several epithelial tissues, is regulated by inflammatory cells and fibroblasts that produce a variety of inflammatory signals and ECM molecules, including collagens, laminins, elastin and tenascins [3]. Injuries in adults can induce the transition of epithelial cells to a mesenchymal phenotype, thus promoting fibrosis in various organs. Fibroblasts and myofibroblasts that have differentiated from epithelial cells can be detected in these tissues [15]. The origin of resident myofibroblasts during organ formation is unquestionable due to their hypothetical mesodermal origin. Their origin in organ fibrosis continues to be a topic of intense debate [16]. The origin of scar-producing myofibroblasts has been controversial as to whether α-SMA-positive matrix-producing myofibroblasts are originated from resident fibroblasts, circulating bone marrow-derived fibrocytes or transition from either epithelial or endothelial cells. In kidney fibrosis, the latest findings reported that resident fibroblasts and bone marrow-derived fibrocytes are the major sources of myofibroblasts in kidney fibrosis [17].

Wound healing is a normal biological process in the human body and is achieved through four stages: haemostasis, inflammation, proliferation and remodelling.

Many factors can affect the stages of wound healing and lead to an impairment of normal wound healing. For example, wounds commonly enter into a state of pathological inflammation and scar development associated with fibrosis due to a postponed, incomplete or uncoordinated healing event. Despite recent advances in novel clinical approaches for abnormal wound healing responses in pulmonary disease, therapies for the most common form of pulmonary fibrosis are essentially ineffective, and the pathogenetic signalling pathways leading to the abnormal wound responses are yet to be determined [18]. Furthermore, abnormal wound healing likely enhances the development of hypertrophic scars and keloids. Although advances have been made in the prevention and management of hypertrophic scars and keloids, the mechanism underlying scar and keloid formation has not been fully clarified. However, previous studies into the role of the EMT in development, wound healing, stem cell regulation, fibrosis and tumorigenesis have improved our knowledge of the pathophysiology of hypertrophic scarring and keloids [19].

The numerous molecules and cells implicated in EMT during wound healing highlight the complexity of tissue repair mechanisms. To date, tissue engineering has focused its attention on the crucial role of EMT in successful wound healing. Although some regenerative mechanisms are still unclear, the presence of a mesenchymal cell phenotype resulting from EMT is an essential prerequisite for proper tissue regeneration [20,21].

In this review, we described the EMT programs during physiological and pathological processes focusing in particular on the role played by type-2 EMT in wound healing, fibrosis and tissue regeneration, as well as some latests advancements in the EMT and tissue regeneration field including the modulation of EMT by biomaterials.

## 2. Wound Healing

Wound healing is a multistage dynamic process including haemostasis, inflammation, cell proliferation and tissue remodelling.

The haemostasis phase occurs immediately after injury and results in the formation of a provisional wound matrix [22]. To prevent exsanguination, vasoconstriction occurs and platelets undergo activation, adhesion and aggregation at the site of injury. The key glycoproteins released from the platelet alpha granules include fibrinogen, fibronectin, thrombospondin and von Willebrand factor [23]. As platelet aggregation proceeds, clotting factors are released, resulting in the deposition of a fibrin clot at the site of injury. Near to the damaged area, platelet alpha granules release pro-inflammatory cytokines such as Transforming Growth Factor-α (TGF-α), TGF-β, Epidermal Growth Factor (EGF), Fibroblast Growth Factor (FGF), Platelet-derived Growth Factor (PDGF) and Vascular Endothelial Growth Factor (VEGF) [24]. PDGF is a chemotactic factor which promotes neutrophils migration to the wound site for eliminating contaminating bacteria. With the help of TGF-β, monocytes are transformed to macrophages, which play a key role in increasing the inflammatory response and tissue debridement. Macrophages start the formation of granulation tissue and secretion of various proinflammatory cytokines as IL-1 and IL-6 and growth factors as FGF, EGF, TGF-β and PDGF. Due to the release of VEGF and FGF by platelets, endothelial cells proliferate, resulting in angiogenesis initiation. This event is of vital importance for the synthesis, deposition and organization of a novel ECM. FGF, TGF-β and PDGF then allow fibroblast infiltration. Moreover, TGF-β and PDGF begin phenotypic modifications, transforming fibroblasts into myofibroblasts [25]. Neutrophils, monocytes and macrophages are the fundamental cells of the inflammatory phase [26].

Neutrophils are crucial for eliminating the microbes and cellular debris in the wound site; they release inflammatory factors such as IL-1, IL-6 and TNF-α, and also produce molecules such as proteases and Reactive Oxygen Species (ROS), which may contribute to tissue damage [27].

Macrophages are responsible for the clearance of apoptotic cells, including the removal of dying neutrophils, which terminates the inflammatory response. As inflammation occurs, macrophages undergo a phenotypic transition to a reparative state that activates keratinocytes, fibroblasts and endothelial cells to induce angiogenesis that restores tissue integrity [28]. Angiogenesis, growth of new blood vessels, supply essential nutrients and oxygen to the damaged tissues and play a crucial part in wound healing [29,30]. Macrophages positively regulate the transition to the proliferation phase of healing [31,32].

The third event in wound healing is the proliferation phase. The proliferation stage is characterized by epithelial proliferation and migration over the provisional matrix within the wound, which is referred to as re-epithelialization [33]. The main events during this phase are the substitution of the provisional fibrin matrix with a novel matrix of collagen fibres, proteoglycans and fibronectin to renew the structure of the tissue and help regain its function [34,35].

Once the wound is closed, the immature scar can proceed to the final remodelling phase. The remodelling phase can last up to a year, depending on the severity of the wound [36]. The wound also undergoes physical contraction through the complete wound healing event, which is considered to be orchestrated by contractile fibroblasts (myofibroblasts) that emerge in the wound [37,38].

In pathological wound healing, however, myofibroblasts activity persists and drives tissue alterations, which is particularly evident in hypertrophic scars developing after burn injury and in the fibrotic phase of scleroderma [39]. Myofibroblasts-generated contractions are also typical for fibrosis, affecting vital organs such as the liver [40], heart [41], lung [42,43] and kidney [44].

## 3. Fibroblasts and Myofibroblasts

Fibroblasts migrate into the wound in response to various soluble mediators, released firstly by platelets and successively by macrophages. The migration of fibroblasts in the ECM is regulated by their interaction with ECM components such as collagens, fibrin and fibronectin. Fibroblasts in regular dermis are usually quiescent and sporadically dispersed, while in the temporary matrix of the wound site and in the granulation tissue, they are moderately active and abundant [45]. Injury to blood vessels provokes a rapid haemostatic response and transitions to the inflammatory stage. Inflammatory cytokines such as TGF-β1 and PDGF encourage fibroblast proliferation and migration to the wound site and promote phenotypic modifications transforming fibroblasts into myofibroblasts. Furthermore, their migration and enlargement in the wound site force them to generate and release proteases to open a route for their passage from the ECM into the wound site. The enzymes released by the fibroblasts contain Matrix Metalloproteinases (MMPs), which could be classified as collagenases (MMP-1), gelatinases (MMP-2 and MMP-9, which degrade the gelatine substrates) and stromelysin (MMP-3, which has multiple protein substrates in the ECM) [46].

At the end of proliferative phase, fibroblasts are differentiated into contractile myofibroblasts which mediate wound contraction (Reinke and Sorg, 2012). Myofibroblasts play a vital role in normal tissue repair events, particularly in the skin, where they were first described. During normal tissue repair, they appear transiently and are then lost via apoptosis. Despite this, the chronic presence and persisted activity of myofibroblasts characterize several fibrotic pathologies in the skin and internal organs, including the liver, kidney and lung [47]. Myofibroblasts were detected in the granulation tissue, and their crucial role in wound contraction was reported [48,49].

Initially, fibroblasts with smooth-muscle cell-like characteristics were defined as myofibroblasts [50]. More recent studies have provided a more intricate definition of these cells: myofibroblasts have been described as cells with stellate or spindle morphology, and processing a weakly eosinophilic but conspicuous cytoplasm [51]. Immunohistochemically, myofibroblasts were positive for Vimentin, α-SMA, non-muscle myosin and Extra Domain A (EDA) cellular fibronectin (EDA-FN), and ultrastructurally, these cells show a very developed, rough endoplasmic reticulum, Golgi apparatus, myofilaments with focal densities and gap junctions [51,52].

**Figure 2:**
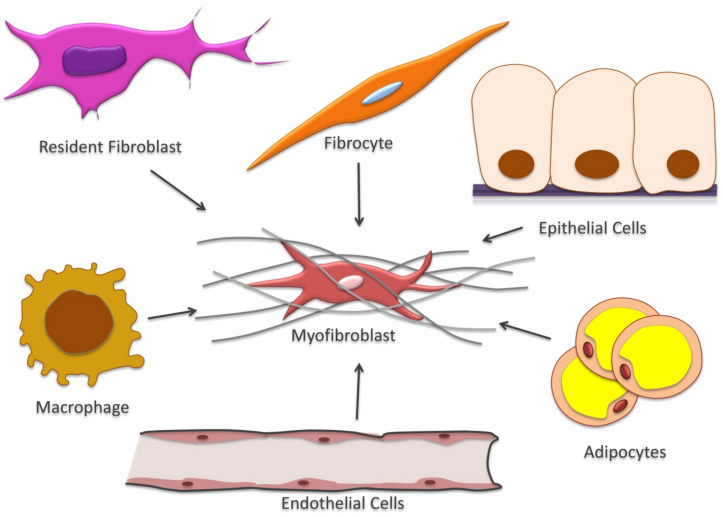
Myofibroblasts multicellular origin. Myofibroblasts can originate from diverse cell population as resident fibroblasts, fibrocytes, adipocytes, monocytes, mesenchymal cells and epithelial/endothelial cells.

Myofibroblasts can originate from several cell types, including resident fibroblasts, fibrocytes, adipocytes, monocytes, mesenchymal cells and epithelial/endothelial cells [53] (Figure 2).

Myofibroblasts act as a double-edged sword and must preserve an adequate equilibrium. From one side, they support the normal wound healing, but from the other, their excessive action can lead to unfavourable contracture and scarring [54].

In physiological tissue repair, myofibroblasts are either naturally lost through apoptosis or become dedifferentiated into fibroblasts as the healing progresses. When these myofibroblasts persist in a closed wound, a hypertrophic scar may be produced [55]. The hypetrophic scar is characterized by hypervascularization and aberrant deposition of ECM molecules [56,57]. In the event of hypertrophic scarring, keratinocytes are excessively differentiated and release fibrotic factors to stimulate fibroblasts, such as VEGF, EGF, Connective Tissue Growth Factor (CTGF, a central mediator of tissue remodeling and fibrosis) and TGF-β [58]. Overabundant collagen accumulation is the principal histopathological feature of hypertrophic scars. Hypertrophic scars contain more type III collagen, fibronectin and hyaluronic acid, all characteristic of the early phases of wound repair and they appear more vascular. Moreover, staining for α-SMA within fibrocytic cells evidenced the presence of this contractile protein in the characteristic collagen nodules of hypertrophic scars [59].

Another function of myofibroblasts is the production of ECM components, such as collagen types I–VI and XVIII, glycoproteins and proteoglycans and the augmentation of matrix elements such as laminin, glycosaminoglycans and hyaluronic acid. However, these cells also generate proteins related to matrix remodelling such as MMPs and tissue inhibitors of metalloproteinases [60].

Both the significance of myofibroblasts in instigating fibrosis in internal organs and the skin (hypertrophic scars) and the persistence of myofibroblasts seem to have a key role in tumour development and spread. This indicates that the eventual downregulation of myofibroblasts and the possible regulation of myofibroblast removal through apoptosis could represent an interesting topic to be further investigated [61].

Fibroblasts and myofibroblasts cooperate in the healing process. It is therefore necessary to understand how myofibroblasts act as contractile cells and how mechanical forces, both involved in wound closure, are modulated [62,63]. This phenomenon is extremely important due to the dual effects that fibroblasts/myofibroblasts have in wound healing. Even a subtle amount of force generation and matrix deposition is beneficial for wound healing, whereas excessive force and matrix production results in tissue scarring and even malfunction of the repaired tissues [62].

## 4. Epithelial to Mesenchymal Transition (EMT)

**Figure 3:**
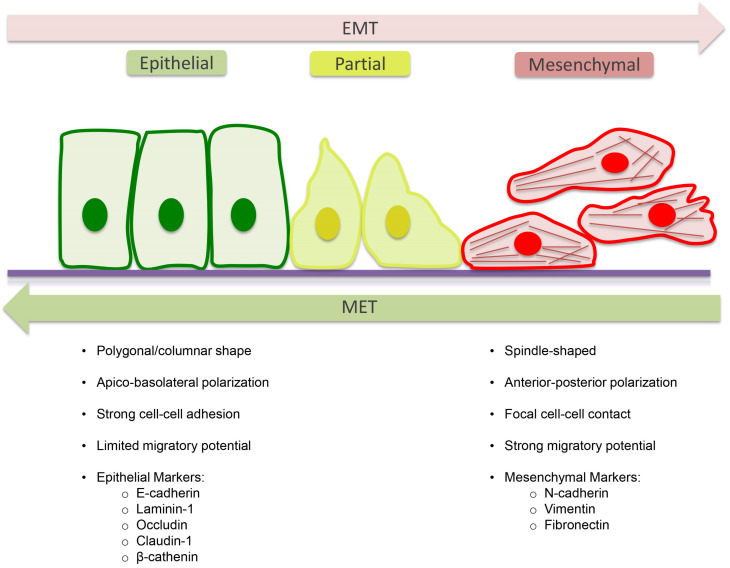
EMT and MET. Schematic view of EMT/MET and the main related molecules. Reprinted from Dongre, A.; Weinberg, R.A. New insights into the mechanisms of epithelial-mesenchymal transition and implications for cancer. *Nat. Rev. Mol. Cell Biol.* **2019**, *20*, 69–84 [1].

Type-2 EMT is involved in fibrotic events, where specialized epithelial cell populations give rise to myofibroblasts with profibrotic and pro-inflammatory activity [64] and in wound healing processes. The final step of the transition to a mesenchymal phenotype involves the formation of the spindle-shaped myofibroblast, which expresses α-SMA and Vimentin but does not express epithelial markers, such as E-cadherin and Zonula Occludens-1 (ZO-1) [65] (Figure 3).

MicroRNAs (miRNAs) act in post-transcriptional regulation of gene expression and are involved in the regulation of skin fibrosis, including TGF-β signaling, fibroblasts proliferation and differentiation, ECM deposition and in EMT process.

Several studies showed diverse expression profiles of miRNAs between hyperplastic scars and normal skin, and the modified miRNAs expression in abnormal scarring could be correlated with TGF-β signaling [66]. Gregory et al. reported the presence of an autocrine TGF-β/ZEB/miR-200 signaling regulatory network that control the plasticity between the epithelial and mesenchymal states of the cells [67]. This control occurs via repression of the translation of specific regulatory proteins, such as E-cadherin, and transcriptional repressors, such as Zinc finger E-box-binding homeobox1 (ZEB1) and ZEB2 [68]. The EMT process during fibrosis is regulated by several cytokines and growth factors, of which TGF-β1 has been shown to play an important role as one of the primary inducers. Furthermore, the mesenchymal state is correlated with migratory ability. All of these features are increasingly being recognized as the primary pathogenic factors of tissue fibrogenesis after damage [10].

The renal expression of TGF-β1 was shown to be elevated in human diabetic nephropathy and TGF-β1 was found to be associated with impaired renal function. Importantly, targeted disruption and inhibition of TGF-β1 signalling protects against renal tubule interstitial fibrosis and EMT [69]. The histological characteristics and regulatory mechanisms of fibrosis are similar across different organs [35]. During physiological wound healing, myofibroblasts disappear via apoptosis in parallel to the epithelialization stage of the restoration [49]. Nevertheless, during pathological scarring, myofibroblasts produce a collagen-rich stiff scar, which ruins the architecture of tissues and modifies the biochemical and biophysical microenvironment, causing an abnormal tissue. In the long term, deregulated activity of myofibroblasts affects tissue function and leads to organ breakdown [69].

## 5. Molecular Mechanism of EMT

Specialized cell surface protein which form epithelial cell–cell junctions are essential for epithelial integrity. Cells are in contact with each other through subapical tight junctions, adherens junctions and desmosomes.

The key events of EMT are the disruption of epithelial cell–cell contacts such as tight junctions, adherens junctions, desmosomes and gap junctions; loss of apical–basal polarity and acquisition of a front–rear polarity; the epithelial actin architecture reorganization and cells acquire motility and invasive capabilities; the repression of epithelial genes and the concomitant activation of mesenchymal gene expression. The MET event allows the cells that have undergone EMT to return to the epithelial state [70,71].

It is largely reported that EMT is regulated by various transcriptional factors such as Snail Family Transcriptional Repressor SNAIL1 and SNAIL2, zinc-finger E-box-binding (ZEB)1 and ZEB2 and TWIST transcription factors that suppress epithelial marker genes and activate genes related with the mesenchymal phenotype. These transcriptional factors act as E-cadherin repressors and play a pivotal role in development, fibrosis and cancer [72].

Several signalling pathways collaborate in the beginning and advancement of EMT and they can promote SNAIL1 expression. SNAIL can be activated by TGF-β and Wnt family proteins, Notch, and growth factors that act through receptor tyrosine kinases (RTKs). For example, SNAIL1 and SNAIL2 cooperate with the SMAD3–SMAD4 complex to promote the TGFβ-mediated repression of E-cadherin and occludin expression. As SNAIL, TWIST expression can be activated by different signaling pathways during development and tumorigenesis. Especially under hypoxic conditions, the transcription factor hypoxia-inducible factor 1α (HIF1α) promotes TWIST expression inducing EMT and tumor cell dissemination [73].

Similar to SNAIL and TWIST, ZEBs work as transcriptional repressors and activators. ZEB expression promotes the activation of SNAIL expression, which consists in SNAIL1 that target the ZEB1 gene. Moreover, TWIST1 co-operate with SNAIL1 to induce ZEB1 expression. ZEB expression is induced in response to TGF-β and Wnt proteins and growth factors that activate RAS–MAPK signalling [74]. Furthermore, EMT can be also regulated by non-coding miRNAs that selectively bind mRNAs [75]. During EMT, the downregulation of miR-200 expression promotes the increase of ZEB1 and ZEB2 levels and EMT advancement.

The EMT process can be regulated by various growth and differentiation factors such as TGF-β, EGF, FGF, hepatocyte growth factor (HGF) and the signalling pathways Wnt and Notch.

TGF-β1 in particular plays a critical role in type-2 EMT.

After birth, TGF-β1 promotes EMT in wound healing, fibrosis and cancer. For example, increased levels of TGF-β1 have been linked to EMT in mesangial cells before kidney fibrosis and fibroblasts from patients with pulmonary fibrosis. Furthermore, the addition of TGF-β1 to cultured renal epithelial cell lines lead to epithelia change from cuboidal to fusiform shape and the acquisition of myofibroblastic mesenchymal nature [76].

Epithelial plasticity responses are linked to the tissue and signalling context and are characterized by a variety of alterations and transitions. The initiation and progression of EMT include different signaling pathways and signaling crosstalk.

## 6. Tissue Regeneration and EMT

Wound healing and tissue regeneration are physiological mechanisms necessary to restore damaged tissues or organ functionality and morphology. In particular, regeneration is the ability to entirely or partly repair the damaged or lost tissues and organs [77,78]. The mechanisms involved in regenerative processes are still unknown. Several studies have reported that regeneration could be related to the proliferation of resident stem cells and duplications of endogenous mature cells [79,80]. In addition, regenerative mechanisms seem to be related to tissue and injury classification, wound size, animal species and developmental stage of the organism [81,82].

In the damaged areas of others soft tissues, parenchymal tissue is progressively replaced by abundant ECM that may cause tissue fibrosis and thus progressive loss of function of the tissue [83,84]. Hypertrophic scars and keloids are the result of excessive ECM deposition. Hypertrophic scars are generated after traumas or surgery interventions and regress in a few months. On the other hand, in people with specific genetic predispositions, injuries can promote the formation of keloids, which are structures that are unable to be spontaneously reabsorbed [85].

The majority of pathological formations are treated with specific therapies. Treatments with antibodies and small molecules directed against scar promoters, such as TGF-β or other cytokines have previously been proposed [86]. In the last years, several researchers have focused their attention on the development of novel treatments that mimic the biological signalling involved in the regenerative processes [87,88].

Over the years, many studies have emerged concerning the biomedical applications of cell migration towards damaged sites and homeostatic mechanisms involved in regeneration and tissue repair [89,90]. In these studies, trans-differentiation processes that take place during tissue repair, tumorigenesis and organogenesis have been widely explored through three-dimensional (3D) tissue-derived spheroid systems. In contrast with 2D structures, 3D systems mimic patho-physiological processes, allowing cell–cell, cell-matrix and cross-talk interactions [91,92,93]. Studies on 3D systems have shown that trans-differentiation processes such as EMT and MET, both of which are involved in regenerative process, can balance stemness of tumoral and non-tumoral cells and promote the differentiation of specific cell phenotypes [94,95].

Interestingly, natural molecules such as honey, curcumin, *Olea europaea* and *Paeonia lactiflora* demonstrated the capability to modulate EMT in preventing illnesses such as pulmonary fibrosis, liver fibrosis, renal fibrosis and cancer. These natural products’ compounds are bioactive elements isolated from natural sources (plants) that can regulate the EMT through anti-inflammatory, anti-fibrotic or antioxidant mechanisms [96,97,98].

Concerning the role of EMT in fibrosis, recent evidence had confirmed that transitional proteins present in epithelial cells are associated with the improvement of fibrosis. As a result, tissue engineering is currently focusing its attention on anti-EMT strategies as promising anti-fibrotic therapies [10].

## 7. Modulation of EMT by Biomaterials

Biomaterials present the capability to promote or prevent EMT in a highly regulated manner, permitting the modulation of EMT event. Biomaterials’ features, such as form, surface topography, wettability and crosslinking capacity, influence their functions [99]. In particular the biochemical and biophysical characteristics can regulate the local tissue microenvironment by modulating the immune system from scarring to total regeneration [100]. Potential approaches require the design of materials with controlled moduli, gradients of ECM proteins and/or soluble factors, multifactiorial strategies utilizing different mechanics, ECM components and soluble factors [28]. All these aspects represent a starting point to take into consideration for the design of novel materials implicated in the modulation of EMT in regenerative medicine and tissue engineering [101]. For example, polyacrylamide (PA) hydrogels, which are synthetic hydrogel matrices with an adjusted stiffness, represent a valuable platform to modulate the EMT event and for evaluating the molecular mechanisms controlling EMT.

It is well known that a rise in the thickness of collagen fibers can be correlated with some diseases, such as fibrosis. Anitha Ravikrishnan et al., in 2016, reported that micro/nano fibrous scaffold to reproduce a proper environment for evaluating the key events leading EMT, demonstrating that the nanofibrous scaffolds represent a valuable tool for investigating EMT during pathology advance [102]. Based on the literature, graphene derivatives are capable to promote lung fibrosis in vivo. In a study reported by Lia et al., in 2018, it was reported that reduced graphene oxide induced EMT activation in A549 cells via a mechanism that involves epithelial markers downregulation and mesenchymal markers upregulation, raising cell migration and invasion capacities [103].

Furthermore, in a work published by Christine-Maria Horejs et al., in 2017, a biomaterial-based approach to target tissue fibrosis in vitro was described: they interface epithelial cells with a cryptic fragment of the laminin b1-chain displayed by the action of MMP2 to trigger a inhibition of MMP2 activity, the gene and protein expression of EMT-related molecules and the morphological alterations linked with fibrosis [104]. Even if the comprehension of molecular events represents a crucial part for the modulation of EMT, the features of the materials are essential factors to take into consideration to regulate EMT process. Thus, in the tissue engineering field are necessary emerging strategies and solutions to prevent or decrease the undesirable side effects of biomaterials.

## 8. Conclusions

In the current review, we report an overview of EMT process with particular interest on type-2 EMT in wound healing, fibrosis and tissue regeneration, as well as some recent progresses in the EMT and tissue regeneration field including the modulation of EMT by biomaterials. The remarkable role of type-2 EMT in tissue repair during various chronic inflammatory disorders has attracted a lot of interest in the scientific community, representing a promising target to be further investigated in tissue renewal, wound healing and fibrosis.

## Data Availability

Not applicable.
